# Supplementary material for: Hypertriglyceridemia Is Associated with More Severe Histological Glomerulosclerosis in IgA Nephropathy
Source: J Clin Med. 2021 Sep 18;10(18):4236. doi: 10.3390/jcm10184236 (PMC8493798; doi:10.3390/jcm10184236)
Supplement: Supplementary file 1 [file jcm-10-04236-s001.zip › jcm-1326083-supplementary.pdf]

Supplementary Materials

Table S1. The Oxford MEST classification of TG groups.

		**TG Group1** <150	**TG Group2** ≥150	*p*-value
M	0	10 (17.5)	6 (10.5)	0.915
	1	25 (43.9)	16 (28.1)	
E	0	28 (49.1)	13(22.8)	0.087
	1	7 (12.3)	9 (15.8)	
S	0	27 (47.4)	14(24.6)	0.269
	1	8 (14.0)	8(14.0)	
T	0	25(43.9)	14(24.6)	0.300
	1	9(15.8)	5(8.8)	
	2	1(1.8)	3(5.3)	

Abbreviations: Oxford classification: M; mesangial hypercellularity, E; endocapillary proliferation, S; segmental sclerosis, T; tubular atrophy/interstitial fibrosis.

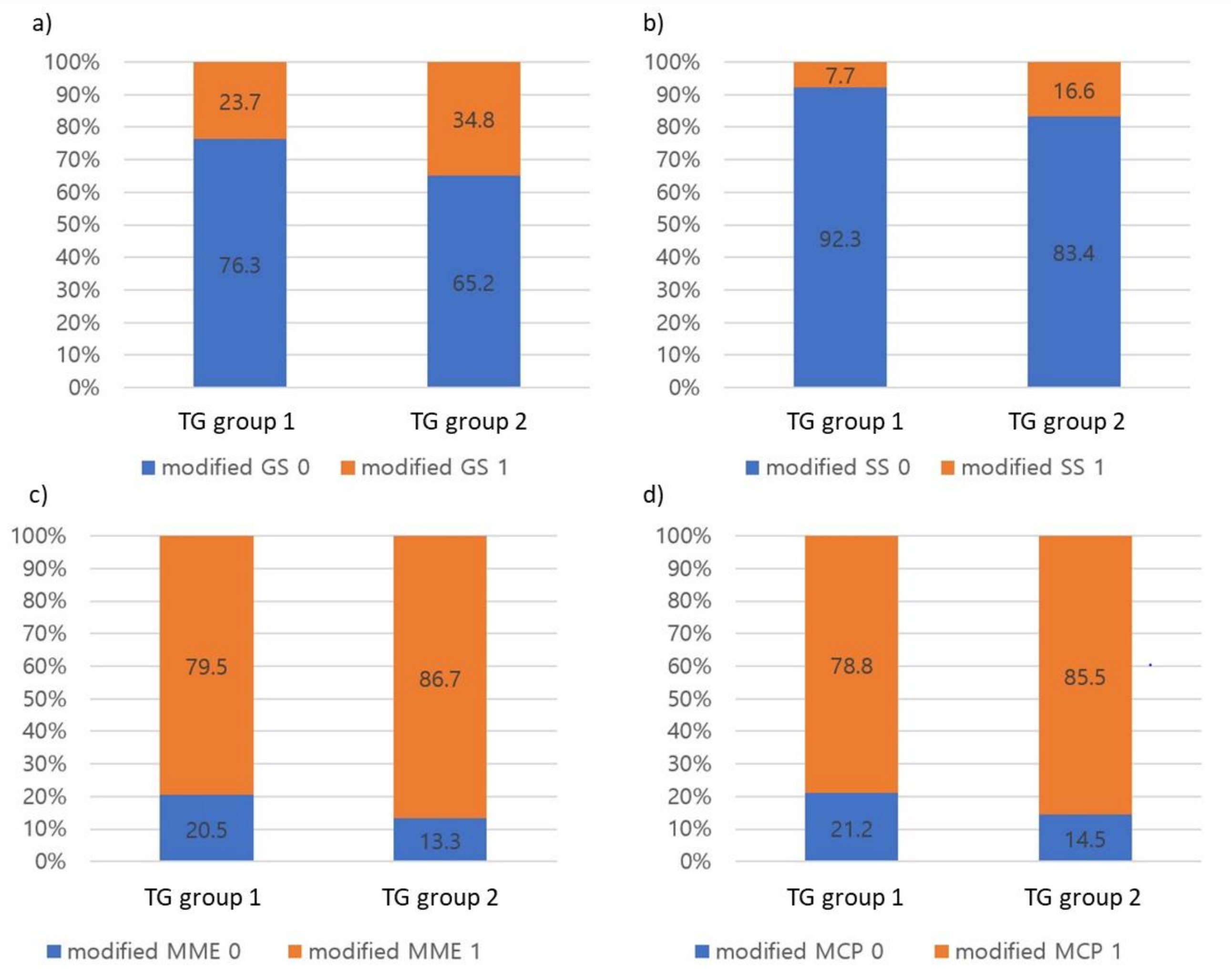

Figure S1. The distribution of modified scores of global sclerosis (GS), segmental sclerosis (SS), mesangial matrix expansion (MME), mesangial cell proliferation (MCP). (**a**) Distribution of modified GS scores for TG groups, (**b**) Distribution of modified SS scores for TG groups, (**c**) Distribution of modified MME scores for TG groups, (**d**) Distribution of modified MCP scores for TG groups.

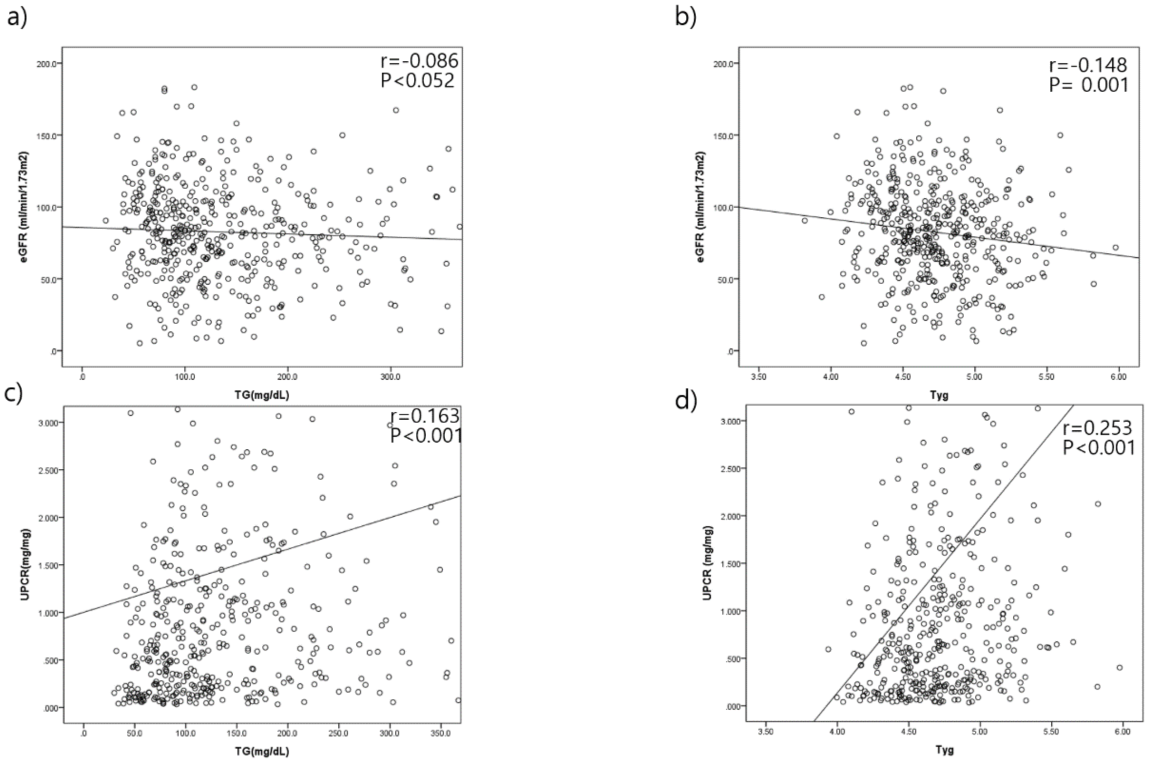

Figure S2. Pearson correlation graph TG and TyG with eGFR and Urine P/Cr. (**a**) Correlation graph between TG and eGFR (r = −0.086, $p < 0.052$), (**b**) Correlation graph between TyG and eGFR (r = −0.148, $p = 0.001$), (**c**) Correlation graph between TG and UPCR (r = 0.163, $p < 0.001$), (**d**) Correlation graph between TyG and UPCR (r = 0.253, $p < 0.001$).
